# Programmed Cell Death Pathways in the Pathogenesis of Idiopathic Inflammatory Myopathies

**DOI:** 10.3389/fimmu.2021.783616

**Published:** 2021-11-24

**Authors:** Jia Shi, Mingwei Tang, Shuang Zhou, Dong Xu, Jiuliang Zhao, Chanyuan Wu, Qian Wang, Xinping Tian, Mengtao Li, Xiaofeng Zeng

**Affiliations:** ^1^ Department of Rheumatology and Clinical Immunology, Peking Union Medical College Hospital, Peking Union Medical College & Chinese Academy of Medical Sciences, Key Laboratory of Rheumatology & Clinical Immunology, Ministry of Education, Beijing, China; ^2^ National Clinical Research Center for Dermatologic and Immunologic Diseases (NCRC-DID), Ministry of Science & Technology, Beijing, China

**Keywords:** idiopathic inflammatory myopathy (IIM), programmed cell death (PCD), apoptosis, autophagy, NETosis, pyroptosis

## Abstract

Idiopathic inflammatory myopathy (IIM) is a heterogeneous group of acquired, autoimmune muscle diseases characterized by muscle inflammation and extramuscular involvements. Present literatures have revealed that dysregulated cell death in combination with impaired elimination of dead cells contribute to the release of autoantigens, damage-associated molecular patterns (DAMPs) and inflammatory cytokines, and result in immune responses and tissue damages in autoimmune diseases, including IIMs. This review summarizes the roles of various forms of programmed cell death pathways in the pathogenesis of IIMs and provides evidence for potential therapeutic targets.

## Highlights

- Dysregulated cell death in combination with impaired elimination of dead cells, get involved in the pathogenesis of IIMs.
- Programmed necrosis, such as NETosis and pyroptosis, seems to play a more important role in the pathogenesis of IIMs, which contribute to the release of autoantigens, damage-associated molecular patterns and proinflammatory cytokines.
- The mechanisms of PCD seem to vary among different subtypes of IIM and require even more precise studies according to different myositis-specific antibodies.

## Introduction

Idiopathic inflammatory myopathy (IIM) is a heterogeneous group of acquired, autoimmune muscle diseases characterized by production of a spectrum of autoantibodies [including myositis-specific autoantibodies (MSAs) and myositis-associated autoantibodies (MAAs)], aberrant regulation of inflammatory responses, and tissue damage of different organs. The most common subtypes of IIMs are represented by dermatomyositis (DM), polymyositis (PM), inclusion body myositis (IBM), immune-mediated necrotising myopathy (IMNM), antisynthetase syndrome (ASSD) and overlap myositis (1). The exact pathogenesis of IIMs has not been fully elucidated, but was reported to be related to genetic and environmental factors, abnormal immune responses and non-immune responses (2).

**Figure 1:**
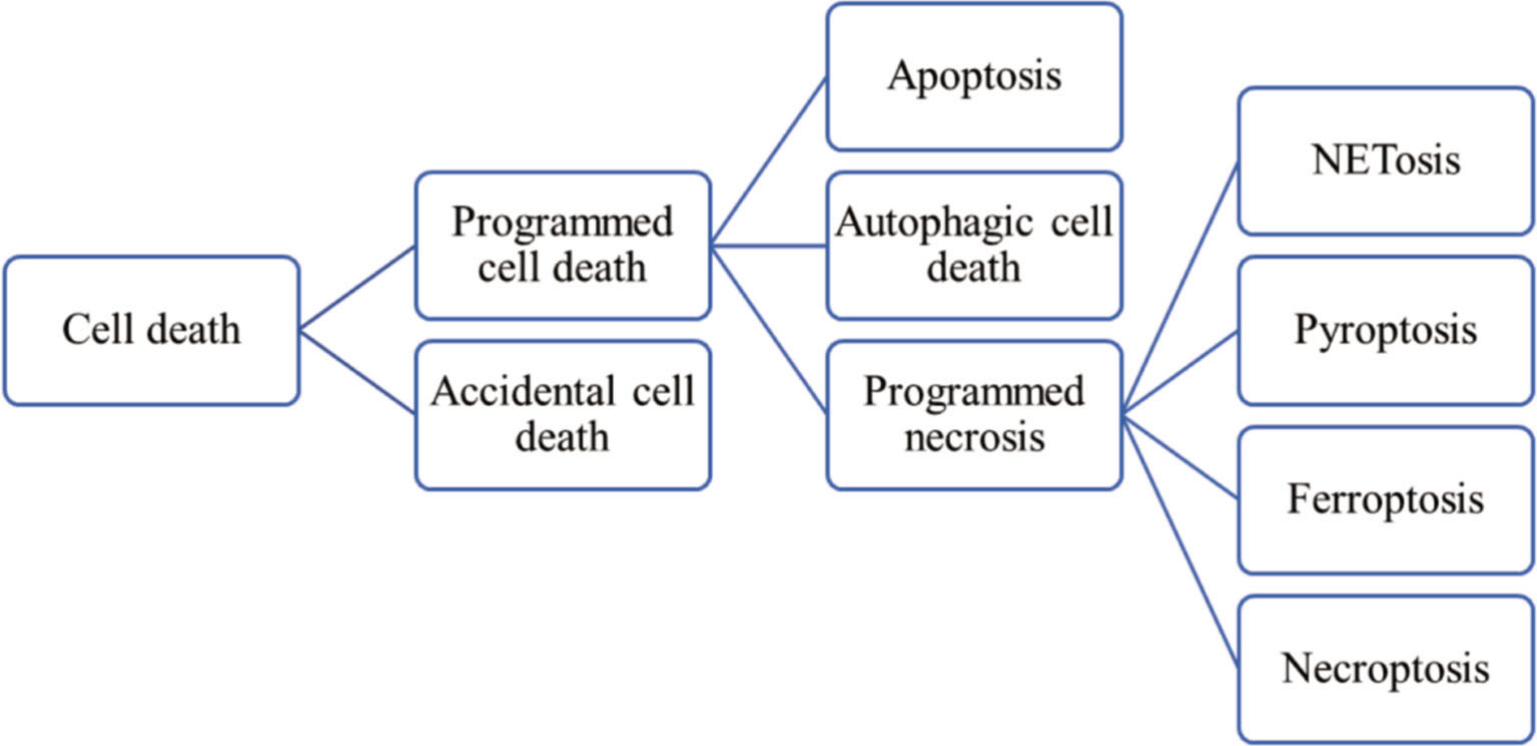
Classification of cell death.

Almost exclusively found in IIM patients, MSAs include antisynthetase autoantibodies (ARS), anti-Mi-2, anti-signal recognition particle (SRP), anti-melanoma differentiation-associated gene 5 (MDA5), anti-nuclear matrix protein 2 (NXP2), anti-transcription intermediary factor 1γ (TIF1γ), anti-small ubiquitin-like modifier activating enzyme (SAE), and anti- 3-Hydroxy-3-methylglutaryl CoA reductase (HMGCR). Their targeting antigens are ubiquitously expressed and are involved in key cellular processes, including gene expression and developmental regulation (3), but how these intracellular components get exposed to the immune system, elicit immune responses and lead to the generation of MSAs, remain unclear. Besides, overexpressed cytokines have been found in the serum and diseased muscle tissues of IIM patients. For instance, the level of type I interferon (IFN) is increased significantly in the muscles of DM patients (4). Therefore, it is reasonable to speculate that abnormal cell death may play a role in the pathogenesis of IIMs. Currently, accumulating evidences have revealed that excessive cell death in combination with impaired elimination of dead cells and debris contribute to the release of autoantigens, danger-associated molecular patterns (DAMPs) and proinflammatory cytokines, and consequently, the over-activated immune and inflammatory responses in IIMs (5). According to functional aspects, cell death can be classified into programmed cell death (PCD) and accidental cell death (ACD) (6). Used to be mistaken for the synonyms of apoptosis, essentially PCD also incorporates autophagy-dependent cell death and programmed necrosis (7) (such as NETosis, pyroptosis, ferroptosis and necroptosis) (**Figure 1**).

In this review, we focus on recent research progression of PCD pathways in the pathogenesis and progression of IIMs to provide evidence for potential therapeutic targets.

## Apoptosis and Secondary Necrosis after Apoptosis

Apoptosis is a genetically-controlled non-lytic cell death pathway, designed to dismantle and remove senescent and injured cells, thereby preventing unwanted inflammation during development, homeostasis, and infection (8). The main features of apoptosis are cytoplasmic shrinkage, membrane blebbing, chromatin condensation and nuclear fragmentation (6). Two distinct apoptotic signaling pathways, intrinsic (also called the mitochondrial pathway) and extrinsic pathways, have been defined. The extrinsic pathway can be triggered by death factors of the tumor necrosis factor (TNF) family [including Fas ligand (CD95L, FasL), TNF-α, and TNF-related apoptosis-inducing ligand (TRAIL)], while the intrinsic pathway is activated by microenvironmental perturbations including endoplasmic reticulum stress (ERS), reactive oxygen species (ROS), or lack of nutrient support (9). Mitochondrial outer membrane permeabilization (MOMP) is the critical and irreversible step for intrinsic apoptosis, which is regulated by Bcl-2 family members (10). Both apoptotic pathways are mediated by specific sets of caspases which act in cascades, among which caspase-8 and caspase-9 being the initiators for the extrinsic pathway and the intrinsic pathway, respectively. Once activated, either caspase-8 or caspase-9 activates executioner caspase-3 and caspase-7 and leads to apoptosis of the doomed cell (11). Macrophages engulf these dead cells in a process called efferocytosis by recognizing “find me” signals [such as ATP, UTP, sphingosine-1-phosphate (S1P) and CX3CL1 (fractalkine)] and “eat me” molecules (such as phosphatidylserine, oxidized LDL-like molecules and C1q-bound serum proteins) released by these cells (10). However, when apoptotic cells are not engulfed efficiently and timely, they undergo secondary necrosis, which is featured with permeabilization of plasma membranes and release of intracellular contents that may activate the immune system (7).

Peripheral T cell lymphopenia has been reported in some patients with DM, which is associated with the increased Fas-mediated apoptosis of T cells. The overexpression of retinoic acid-inducible gene I (RIG-I) could induce T cell apoptosis, but the mechanism is far from clear (12). Also, lymphopenia may be resulted from the decreased autophagy, which is discussed later below (13). Noteworthily, in patients of IIM, T cells infiltrating in the muscles are dominated by CD28^null^ T cells, partly due to chronic antigenic stimulation (14), which show an increased percentage in peripheral blood mononuclear cells (PBMCs) as well (15–18). CD28^null^ T cells infiltrating in IIM muscles are generally regarded as terminally differentiated and apoptosis resistant with properties of replicative senescence (16, 17, 19, 20), because of decreased expression of Fas (21), and increased expression of antiapoptotic molecule Bcl-2 (22), Bcl-x_1_ and cyclin-dependent kinase inhibitors p16 and p57 (20), upregulation of the phosphoinositide 3-kinase pathway (23) (which inhibits Fas-initiated apoptosis), and upregulation of various inhibitory natural killer cell receptors (iNKRs) (24) (such as CD94/NKG2A). CD28^null^ T cells are not capable of costimulatory interaction with CD80 and CD86, but this does not represent a global loss of costimulatory receptor expression. The upregulation of alternative costimulatory molecules, such as inducible costimulator (ICOS), CD134 and CD137, has been reported in CD28^null^ T cells after CD3 ligation (25). Moreover, this T-cell phenotype is suggested to be treatment-resistant for its persistence in muscle tissue after high doses of glucocorticoids and other immunosuppressive treatment, which is correlated with a poor clinical response (17), and could be due to a significant loss of glucocorticoid receptors (GCRs) (26). Therefore, CD28^null^ T cells are an emerging target of interest for treatment in refractory myositis patients. Existing drugs that could downregulate T cells include calcineurin inhibitors (e.g., tacrolimus and cyclosporine) and abatacept. Calcineurin inhibitors could prevent calcineurin from dephosphorylating nuclear factor of activated T cell (NFAT) proteins and then repress transcription of IL-2, and thereby restraining the differentiation and survival of T cells (27). A single-arm prospective clinical trial revealed that initial combination treatment with tacrolimus and GCs could improve short-term mortality of PM/DM-ILD patients with satisfactory safety (28). Abatacept, an agonist of cytotoxic T-lymphocyte antigen-4 (CTLA-4), could interfere with the activation of T cells by binding CD80/CD86 on the surface of antigen‐presenting cells (APCs). A phase IIb, delayed-start clinical trial of abatacept was conducted in 20 refractory DM/PM patients. Decreased disease activity was observed in 42% of the patients, as well as upregulated Foxp3^+^ regulatory T cells in repeated muscle biopsies (29). There is a phase III, randomized, double-blind trial underway to further evaluate abatacept for myositis treatment (Clinical Trial Identifier NCT02971683).

In PM, CD28^null^ T cells have been demonstrated to be able to exert direct cytotoxicity towards myocytes by polarizing perforin and secreting granzyme B, and indirectly contribute to myotube cell death by releasing proinflammatory cytokines IFN-γ and TNF which could induce surface expression of MHC, rendering the myocytes more sensitive to cytotoxic attacks (30). An *in vitro* study also identified that culturing myoblasts with IFN-γ or TNF alone could upregulate inflammation related transcription factors (NF-κB, nuclear factor-κB) and induce apoptosis (31), implying that extracellular inflammation induces further inflammatory changes and forms a sustained loop of inflammation leading to cell death. One study on IBM showed that the combination of β-amyloid (Aβ) with IFN-γ stimulating pathogenic $\text{N}{\text{O}}_{2}^{-}$ production *via* induction of iNOS gene expression could induce apoptosis of myocytes (32), while the other found Aβ alone is sufficient for myofiber apoptosis (33). In hereditary IBM, mutations in gene GNE could lead to impaired apoptotic signaling, thus causing degenerative process and muscle loss (34). Moreover, the expressions of Fas and cytoplasmic caspase-8 and -3 of myocytes could be upregulated by proinflammatory cytokines as well (35), but whether myocytes apoptosis mediated by Fas/FasL interaction is involved in the pathogenesis of IIM is controversial. Fas expression in muscle fibers has been reported with very different frequencies (36–39). The presence of myocytes with TUNEL positive nuclei has been reported in IIMs but was very rare. As for this phenomenon, some researchers consider that the frequencies of apoptosis are too low to prove the relevance to pathopoiesis, while others believe that it is attributed to the prompt efferocytosis. These rare apoptotic myocytes were surrounded by CD8^+^ T cells and granzyme B^+^ cells with absence of Fas and upregulating MHC-I, favouring a cytotoxic mode of apoptosis induction rather than a Fas-mediated mechanism (37), which is in line with an earlier study, suggesting Fas expression may be attributed to the new gene expression in regenerating fibers (38). The resistance of myocytes to apoptosis is attributable to anti-apoptotic intracellular proteins, such as Bcl-2 (36), FLICE (Fas-associated death domain-like IL-1-converting enzyme)-inhibitory protein (FLIP) (40) and human IAP-like protein (hILP) (41). Notably, in contrast with previous study, Bcl-2 has been reported to exhibit lower expression in diseased muscle compared with normal muscle (39). Therefore, the mode of myocyte death needs to be further investigated.

Nevertheless, some therapeutic strategies regarding apoptosis have been identified. Resistance exercise (RE) has been demonstrated to reduce Aβ accumulation in chloroquine (CQ)-induced rat model of IBM, thus inhibiting mitochondrial-mediated apoptosis of myofibers and improving mitochondrial function through increased mitochondrial biogenesis, upregulated mitophagy, and activated sirtuin 3 signaling (42). Alemtuzumab, a recombinant DNA-derived humanized monoclonal antibody targeting CD52, was also beneficial for IBM patients, as it lowered the count of peripheral and endomysial T cells with reduced mRNA expression of Fas (43). It could enhance apoptosis in B cells by upregulating the expression of caspase-8 and caspase-3 in chronic lymphocytic leukemia as well (44), which may provide a valuable reference for IIM. Besides, pro-senescent interventions consisting of exercise and AMP-activated protein kinase (AMPK) activation induced apoptosis of fibro-adipogenic progenitors (FAP) and promoted muscle regeneration in a murine chronic inflammatory myopathy (CIM) model, suggesting that the FAP-targeted intervention may be therapeutic (45). In PM/DM, expressions of cathepsin B (CB) and calpain are increased in muscle and lung tissues, which promotes cell apoptosis and inflammation. Calpeptin (calpain inhibitor) ameliorated morphological changes of apoptosis in IFN-γ or TNF-α treated myoblasts through both mitochondrial pathway and ERS pathway (31, 46). Also, the administration of CA-074Me, a specific inhibitor of CB, could attenuate apoptosis of myocytes and lung epithelial cells, and reduce lung interstitial inflammation and fibrosis in the guinea-pig model of PM (47, 48).

## Autophagy and Autophagic Cell Death

Autophagy is a highly conserved catabolic and homeostatic process by which subcellular components are secluded and degraded *via* lysosomes under stress conditions, such as ERS, nutritional deprivation, mitochondrial injury, and inflammation (49). It is featured with vacuolization of the cytoplasm and accumulation of double-membraned vacuoles (i.e., autophagosome) in morphology. According to the modes of cargo transferring to the lysosome, autophagy is classified as macroautophagy, microautophagy and chaperone-mediated autophagy (CMA). In addition to elimination of intracellular aggregates and damaged organelles, autophagy plays crucial roles in inflammation and immune-system function, mediating cytoprotective rather than cytotoxic effects. The interplay between autophagy and cytokines is fundamental to modulate inflammatory as well as immune responses. For instance, TNF-α can induce autophagy and in turn, whether autophagy up- or down-regulates TNF-α formation depends on the cellular context (50). Autophagic cell death is a type of PCD that relies upon the autophagic machinery or constituents thereof, with massive autophagic vacuolization of the cytoplasm but without chromatin condensation (51).

Most IIM studies with regard to autophagy focus on IBM. Rare missense variants in autophagy-related genes, such as VCP, HNRPA2B1, BAG3, SQSTM1, FLNC and ZASP have been identified to occur at a higher frequency in IBM patients than in control populations (52). VCP mutations could result in defective myotube formation, increased apoptosis and increased autophagy (53). Moreover, a study based on whole exome sequencing (WES) identified missense variants in FYCO1, which encodes for an LC3-binding protein accumulating at rimmed vacuoles and is implicated in microtubule transport of autophagosomes, were statistically enriched in IBM patients (54). Collectively, these findings revealed a strong tie between IBM susceptibility and autophagy. In IBM muscle tissues, increased formation of vacuolar autophagosomes has been identified along with massive protein aggregation, as indicated by increased levels of p62, LC3, mTOR-mediated phosphorylation of p70SK, α-synuclein and TDP-43 (55–57). These markers could be ancillary tools to differentiate IBM from other IIMs (56). In the inflammatory milieu in muscle, the upregulated proinflammatory cytokines, such as IL-1β (58), TNF-like weak inducer of apoptosis (TWEAK) (59) and TRAIL (60), get involved in stimulating autophagic cell death. Besides, the overexpression of MHC-II in inflamed muscle fibers, partly on account of the increased cytokine TNF-α, could induce autophagy and interact with IFN-γ to translocate intracellular MHC-II to the myocyte surface further (61). Also, defective autophagy could drive increased MHC-I expression because of the weakening ability of MHC-I internalization for degradation (62). All the evidence revealed that dysregulated autophagy might contribute to antigen presentation for MHC-I and II, and maintain the inflammatory response in a vicious circle (63).

The overmuch autophagy reflected by overexpression of autophagic proteins in muscle, and impaired protein degradation, contributing importantly to consequent accumulation of multiprotein aggregates, are key factors in the myofiber degeneration characteristic of IBM (55, 64, 65). Specially, the accumulation of amyloid-β42 oligomers, could cause reduction in muscular peak force and amplitude of Ca^2+^ transients in mouse models of IBM (66), suggesting that their cytotoxicity contribute importantly to IBM pathogenic cascade. Cylindromatosis (CYLD), a deubiquitinating enzyme, co-expressed with autophagy-related proteins in IBM, contributed to muscle damage by attenuating autophagic clearance of protein aggregates (67). Cacciottolo et al. found the upregulation of CMA components in sIBM muscle fibres, which revealed cellular attempts to activate CMA and remove protein aggregates (68). However, this attempted compensation might not fully work because of the decreased activity of proteolytic enzymes in lysosomes (55). Arimoclomol, an inducer of heat shock response, can upregulate chaperone expression, thereby promoting CMA in stressed cells and curbing the formation of protein aggregates. Treatment with arimoclomol ameliorated IBM-like pathology in myoblasts and mutant VCP mice, and it was safe and well tolerated in a proof-of-concept clinical trial of IBM patients (69). To further evaluate the efficacy of this drug in IBM, a multisite phase II clinical trial has been completed (Clinical Trial Identifier NCT02753530). Moreover, RE could facilitate fusion between autophagosomes and lysosomes in IBM animal models, hence improving impaired macroautophagy (70).

In addition to IBM, autophagy activation could also be detected in PM, DM and IMNM muscle tissues, whereas the autophagic activation, modulation and interaction with the immune system, are different in each type of IIM (60, 71, 72). In IBM, lysosomal enzymes Cathepsin B and D, are inhibited, while in PM, their activities were actually increased (55). Dysfunctional CMA (73) and mitophagy (a specific autophagic elimination of mitochondria) (74), were reported to occur in IMNM muscles. The decreased autophagy and increased apoptosis of circulating CD3^+^ T cells have been demonstrated in PM/DM patients, and this phenomenon could be turned around by the treatment of autophagy inducer rapamycin, hence preventing lymphopenia, which suggested that autophagy may play a potential cytoprotective role in PM/DM *via* inhibition of apoptosis in CD3^+^ T cells (13). Intravenous immunoglobulin (IVIG) therapy has been demonstrated to induce autophagy in PBMCs and reduce circulation proinflammatory cytokines in IIM by activating AMPK and inhibiting mTOR phosphorylation, thus mediating anti-inflammatory effect (75).

## NETs and NETosis

Neutrophils are critical immune cells at the frontline of immune defense, responsible for eliminating pathogens by multiple mechanisms, including phagocytosis, production and release of antimicrobial proteins, and formation of neutrophil extracellular traps (NETs) (76). NETs are web-like structures composed of histones, granular proteins and decondensed chromatin, which could be autoantigens and DAMPs to break immune tolerance in predisposed hosts (77–79). NET-derived mitochondrial DNA could induce type I IFN production through the DNA-sensing cGAS-STING pathway in myeloid cells (80). Antimicrobial peptide LL-37 could activate type I IFN as well (81). Also, the components of NETs are detrimental to vessels and muscles (82–86). Citrullinated histones exerted toxic effect to decrease the viability of myotubes (87). Accompanied by the formation of NETs, neutrophils die, which is called the NETosis. Essentially, the internal environment homeostasis of IIM patients is disrupted with abnormal cytokine levels (88), which may generate unexpected NETs formation. These NETs irritate more production of proinflammatory cytokines, maintaining a vicious circle of sustained NETosis. For instance, this lytic process could promote the production of IL-6 and IL-1β in macrophages (89). If excessive NETs cannot be cleared timely and efficiently by DNase I and macrophages, inflammation and autoimmunity will ensue (90).

IIM patients exhibited significant increased NETs, especially in individuals with ILD, which is resulted from decreased activity of DNase I (91). Low-density granulocytes (LDGs), a unique subset of neutrophils with proinflammatory phenotype, are prone to commit NETosis and secrete proinflammatory cytokines (92). LDGs have been reported to display an increased percentage in PBMCs in DM patients, especially those complicated by ILD, along with increased NETs, which may further contribute to the progression of ILD (93). Abnormal regulation of NETs has been reported to be associated with MSAs. An *in vitro* study showed that anti-MDA5 Ab^+^ serum could directly induce NET formation (94). Interestingly, NET levels exhibited a significant rise in patients with anti-MDA5 or anti-TIF1 antibodies, yet not in patients with anti-Jo-1 positive (87). Contradictorily, Zhang et al. found patients with anti-Jo-1 antibodies exhibited lower DNase I activity than those without anti-Jo-1 antibodies (91). Therefore, studies with larger sample sizes are needed to clarify the association between MSAs and NETs. NETosis may be related to prognosis as well. Anti-MDA5 antibody positive and hyperferritinemia have been identified as the poor prognostic factors of DM. The level of serum cfDNA, which is the product of NETosis, was reported to significantly increase in anti-MDA5 Ab^+^ subset and hyperferritinemic subset, hence it may be a potential indicator of prognosis (94).

A recent report described the presence of calcium crystal–induced NETosis in JDM. The engulfment of calcium crystals by tissue-infiltrating neutrophils, triggered NETosis which is NADPH oxidase- and complement–dependent (95). Also, circulating immune complexes may contribute to the elevated NET levels in JDM (95). JDM patients can develop atherosclerosis during progression into adulthood. Such IIM-associated cardiovascular disease may be related to the oxidation of high-density lipoprotein (HDL) through NETs-derived MPO (96).

## Pyroptosis

Pyroptosis is a lytic and proinflammatory form of PCD depending on gasdermin family. Three pathways have been identified, including the caspase-1-mediated canonical pathway stimulated by PAMPs or DAMPs, the noncanonical pathway requiring caspase-4, 5 (for human) or caspase-11 (for murine) triggered by lipopolysaccharide (LPS), and caspase-3-dependent pathway. The best-studied pyroptosis pathway is that mediated by gasdermin D (GSDMD) with downstream of nucleotide-binding and oligomerization domain-like receptor family pyrin-domain containing 3 (NLRP3) inflammasome activation, which can recruit and activate inflammatory caspases. The activated caspase-1 or caspase-4/5/11, cleaves GSDMD and exposes its N-terminal domain, which binds to phosphoinositides in the cell membrane and forms large pores, thus driving cytoplasmic swelling, cytolysis, and release of cellular contents (97). Also, caspase-1 cleaves IL-1β and IL-18 to produce mature cytokines, but whether these cytokines are actively secreted or released *via* pyroptotic membrane rupture remained unclear (98). Other released contents, including cleaved GSDMD, chemokines, ATP and HMGB1, recruit immune cells and expand tissue inflammation (99). Caspase-3, recognized as the apoptotic executioner by convention, has been reported to specifically cleave gasdermin E (GSDME), thereby initiating pyroptosis, and whether cells with caspase-3 activated undergo apoptosis or pyroptosis, depends on the expression level of GSDME (100).

Currently, the three mentioned pathways have all been demonstrated in muscle tissues of IIMs. Liu et al. first reported that the GSDME-dependent pyroptosis got involved in the pathogenesis of perifascicular atrophy (PFA), a pathognomonic histologic feature of DM (101). Soon afterwards, Ma et al. demonstrated the implication of noncanonical pathway in the animal model of experimental autoimmune myositis (EAM) as well, and glyburide and brilliant blue G (BBG) could lower the levels of pyroptotic markers and alleviate symptoms (102). However, these two studies verified pyroptosis by detecting the full length of GSDME or GSDMD, rather than the cleaved forms. Besides, upregulated glycolysis has been reported in the lesioned muscle tissues of DM/PM, which further promoted myocyte pyroptosis by activating the NLRP3 inflammasome and exposing N-GSDMD. Treated IFN-γ-stimulated-myotubes with shikonin, a pyruvate kinase isozyme M2 (PKM2) inhibitor, could mitigate NLRP3 inflammasome activation and suppress pyroptosis (103). Intriguingly, the levels of PKM2 and IL-1β were related to MSAs, and were especially high in patients with anti-SRP autoantibody (103).

## Other Forms of PCD: Ferroptosis and Necroptosis

### Ferroptosis

Ferroptosis is a newly proposed cell death with unique morphological structures and biochemical expressions, caused by oxidative damage due to the excessive accumulation of iron-dependent lipid peroxidation products. It usually shows necrosis-like morphological changes with mitochondrial abnormalities, such as condensed membrane and reduced or absent crista (104). Whether a cell will undergo ferroptosis is linked with many factors, such as its level of polyunsaturated fatty acid (PUFA), iron metabolism, and glutathione (GSH) biosynthesis. The inhibition of cystine-glutamate antiporter (system Xc-) and the inactivation of GSH peroxidase-4 (GPX4) lead to the depletion of cellular GSH, and the impaired clearance of ROS, thus causing collapse of cellular redox homeostasis and accumulation of ROS from lipid peroxidation or Fenton reaction, ultimately resulting in lipid membrane damages and cell death (7). Ferroptotic peroxidation products are powerful inducers of autophagy (e.g., reactive aldehydes) (104), and excessive autophagy promotes ferroptosis in turn, by degrading iron-storage protein ferritin and hence increasing cellular iron concentration (105). This specific autophagic process is called ferritinophagy. Of note, hyperferritinemia is frequently accompanied by IIM-ILD, and is associated with disease severity and prognosis (106). Moreover, mitochondria play a proactive role in cysteine-deprivation-induced ferroptosis by fueling metabolism and lipid ROS production (107). Besides, mitochondrial abnormalities and increased level of ROS, have been reported in IIM, proposed to be vital mediators in IIM pathophysiology (108–110). Taken together, it is logical to hypothesize that ferroptosis is implicated in the development of IIM, so further in-depth studies are necessary to elucidate the exact role of ferroptosis in IIMs.

### Necroptosis

Necroptosis is an inflammatory form of PCD characterized by receptor-interacting protein kinase 3- (RIPK3-) mediated activation of mixed lineage kinase domain-like protein (MLKL) and permeabilization of the plasma membrane (111). Although apoptosis and necroptosis frequently share common triggers, including death receptors and IFN, downstream signaling pathway of these triggers leading to survival, apoptosis or necroptosis depends on the availability of cellular inhibitor of apoptosis (cIAPs), FLIP, or caspase-8 (112). In the absence of caspase-8, the necrosome (i.e., RIPK1/RIPK3 complex) activates necroptotic pathway, thus promoting the recruitment and phosphorylation of MLKL, and then, the activated MLKL translocates to the cell membrane and damages the integrity, leading to the release of cell contents and generating inflammation (113). In addition, necroptosis regulators RIPK3 and MLKL have been reported to play an independent role in inflammation irrespective of cell death – promoting NLRP3 inflammasome activation and IL-1β secretion (114, 115). Collectively, these results indicated that necroptosis can enhance inflammation and may be implicated in the pathogenesis and progression of autoimmune diseases. For instance, necroptosis has been reported to contribute to B-cell lymphopenia in systemic lupus erythematosus (116). IFN-γ could downregulate necroptosis by inhibiting MLKL and cFLIP, thereby exerting protective effects in autoimmune arthritis (117). GSK2982772 is a highly selective inhibitor of RIPK1, while a latest randomized, placebo-controlled study found GSK2982772 no meaningful clinical improvement of RA compared with placebo (118). No studies investigate the association between IIM and necroptosis currently.

## Crosstalk Between PCD Pathways

The PCD pathways are tightly connected and the cross regulation between them is complex. In most circumstances, apoptosis and autophagy are mutually inhibited. Autophagy can reduce the abundance of pro-apoptotic proteins in the cytosol (e.g., caspase-8), while activated caspases can degrade essential autophagic proteins (e.g., BECN1) (119). On the contrary, there is a mutual promotion between ferroptosis and autophagy, which is discussed above in the ‘Ferroptosis’ section.

Different cell death pathways can be activated with the same signal. For example, disturbed redox homeostasis and excessive ROS attributed to sustained activation of ER stress pathway, which is clearly of etiological relevance in IIM (120), could activate all the PCD pathways aforementioned. Moreover, biochemical and cellular consequences of one type of cell death can have profound influence on the activity of another type of cell death (121). GSDMD, the executor of pyroptosis, also plays a crucial role in NETosis. During NETosis, serine proteases released from neutrophil granules could cleave GSDMD, and then activated GSDMD in turn permeabilizes granules to enhance proteases release and promotes nuclear expansion. Further, activated GSDMD forms pores in the plasma membrane, promoting NET release (122).

Various cell death modes can coexist, and cells can switch between one death pathway to another. Caspase-8 is a crucial molecular switch for apoptosis, necroptosis and pyroptosis. It can not only directly cleave caspase-3 to induce extrinsic apoptosis, but also get involved in other cell death pathways. When TGF-β activated kinase-1 (TAK1, cell survival kinase) is inhibited, caspase-8 could cleave GSDMD and induce pyroptosis (123). The loss of caspase-8 or its enzymatic activity could lead to MLKL-dependent necroptosis. If necroptosis is blocked, enzymatic inactive caspase-8 could indirectly activate GSDMD and cause pyroptosis by driving ASC (apoptosis-associated speck-like protein, adaptor protein of NLRP3 inflammasome) speck formation, which leads to caspase-1 activation (124). Overall, increasing evidence points to caspase-8 as a central regulator of cell death, and it promotes apoptosis, necroptosis, or pyroptosis depending on its posttranslational state, the cell type, and the stimuli (125). The intricate crosstalk between pyroptosis, apoptosis, and necroptosis has led to the proposal of PANoptosis. It is regulated by the PANoptosome complex, a molecular scaffold for the contemporaneous engagement of key pyroptotic, apoptotic, and necroptotic machinery (125). PANoptosis has been reported in microbial infection, inflammatory diseases, cancer and cytokine storm. For instance, in COVID-19, increased circulating levels of TNF and IFN-γ synergistically induce PANoptosis characterized by activation of pyroptotic (GSDME), apoptotic (caspase-8/3/7) and necroptotic (pMLKL) molecules, facilitate further pathogenic cytokine release through membrane pores and cell lysis, culminating in a life-threatening cytokine storm (126). Whether PANoptosis occurs in anti-MDA5-associated ILD, which is prone to be complicated by cytokine storm, remains uninvestigated.

Therefore, due to the intricacies and connections between each PCD pathways, which death pathway is dominant, and whether there is synergy and the simultaneous activation of multiple pathways in IIM need to be considered. Taking an integral view of cell death in IIM may improve our understanding of pathogenesis and aid in the development of therapeutics.

## Conclusion

**Figure 2:**
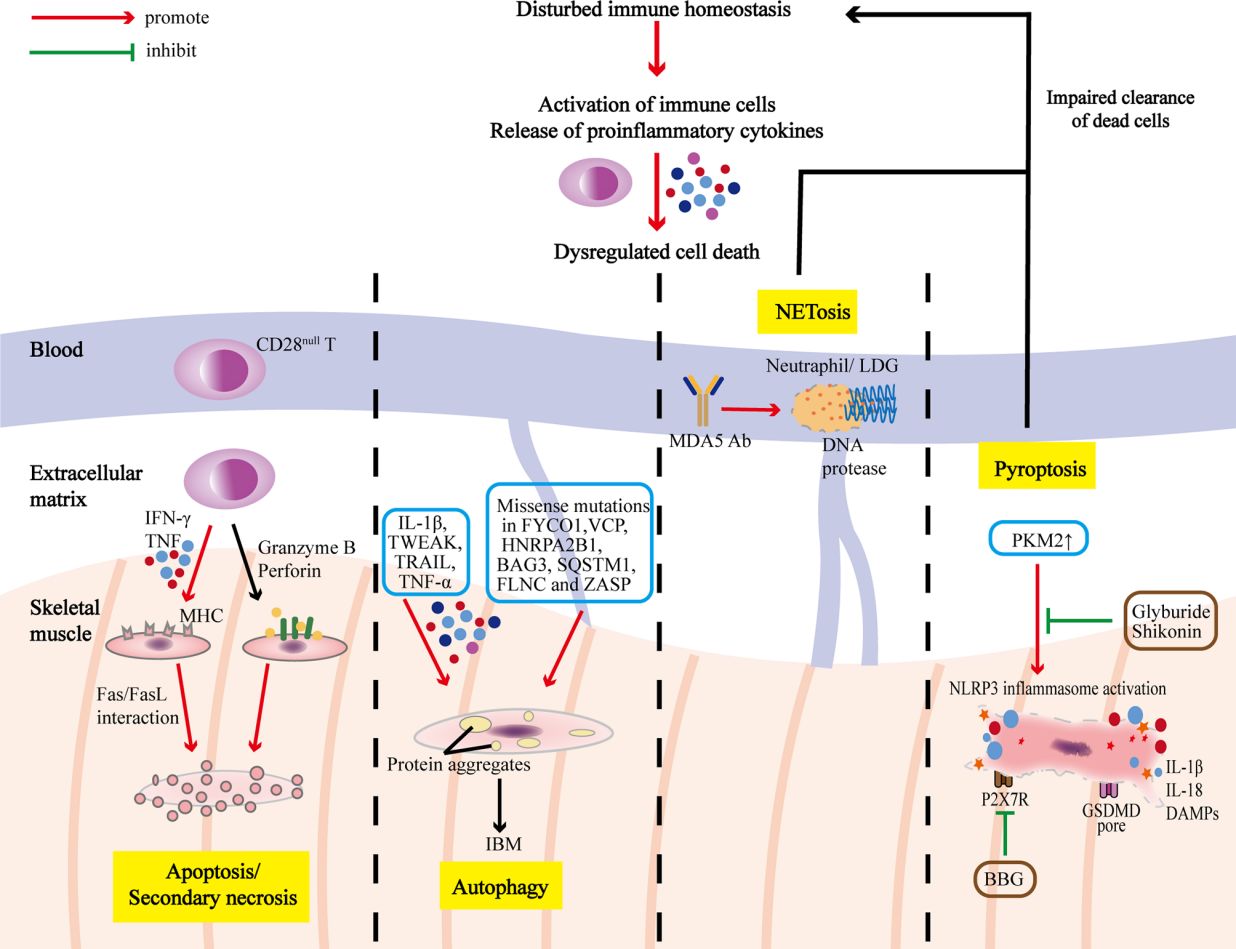
PCD pathways in the pathogenesis and progression of IIMs. The immune homeostasis of IIM patients is disrupted with activation of immune cells and release of proinflammatory cytokines, which could lead to dysregulated cell death. T cells infiltrating in the muscles are dominated by CD28^null^ T cells, which are apoptosis-resistant, and could exert polarize perforin and secrete granzyme B to induce myocyte apoptosis, or release IFN-γ and TNF to induce surface expression of MHC, rendering the myocytes more sensitive to cytotoxic attacks. Overmuch autophagy and impaired protein degradation result in accumulation of multiprotein aggregates, which causes myocyte degeneration characteristic of IBM. Excessive programmed necrosis (such as NETosis and pyroptosis) contribute to the release of proinflammatory cytokines, and DAMPs, and activation of NLRP3 inflammasome, further amplifying immune responses.

Immune and non-immune factors contribute to abnormal cell death in IIMs (**Table 1**), and dysregulation of PCD further amplifies inflammatory responses, playing an important part in the pathogenesis and progression of IIM (**Figure 2**), although it has not been unveiled clearly. Further in-depth studies on these PCD pathways will extend our knowledge on the pathogenic mechanism of IIMs, and targeting different steps to inhibit PCD processes and promoting the clearance of death materials may be promising therapeutic strategies for IIMs (**Table 2**). In addition, the particularity of autoantibodies is noteworthy, as patients with diverse autoantibodies exhibit different clinical manifestations, prognosis, organ involvements, and treatment responses, suggesting that potential immunopathogenic mechanisms may be different. Besides, targeted antigens by MSAs are intracellular components, so we speculate that their exposure to immune system and followed generation of MSAs are attributed to the dysregulation of PCD. Therefore, it’s reasonable to put emphasis on the association of distinct MSAs with PCD pathways in further studies. Also, PCD pathways are intimately linked and interdependent, making it necessary to take a comprehensive approach to investigate PCD pathways.

**Table 1:** Factors inducing abnormal myocyte death in IIMs.

Factors	Death pathways	Effects	Year	References
**Perforin, granzyme B**	Apoptosis	Exert direct cytotoxicity	2016	(30)
**IFN-γ, TNF**	Apoptosis, autophagy	Induce surface expression of MHC	2011, 2016	(30, 31, 61)
**Aβ, IFN-γ**	Apoptosis	Stimulate $\text{N}{\text{O}}_{2}^{-}$ production *via* induction of iNOS gene expression	2000, 2001	(32, 33)
**Mutations in gene GNE**	Apoptosis	Increase caspases-3 and -9 expression	2007	(34)
**IFN-γ, TNF, IL-1β**	Apoptosis	Increase Fas, caspases-3 and -8 expression	2009	(35)
**Mutations in gene VCP**	Autophagy, apoptosis	Lead to impaired protein degradation	2009	(53)
**Missense variants in gene FYCO1**	Autophagy	Lead to impaired microtubule transport of autophagosomes	2017	(54)
**IL-1β, IFN-γ**	Autophagy	Increase phosphorylation of the mitogen activated protein kinases and induce accumulation of amyloid	2017	(58)
**TRAIL**	Autophagy	Induce NF-κB activation and autophagic cell death	2011	(60)
**MSAs**	NETosis	Unclear	2014, 2018, 2020	(87, 91, 94)
**Calcium crystal, immune complexes**	NETosis	Induce formation of NETs	2020	(95)
**PKM2**	Pyroptosis	Activate NLRP3 inflammasome	2021	(103)

iNOS, inducible nitric oxide synthase; Aβ, β-amyloid; VCP, valosin containing protein; FYCO1, FYVE and coiled-coil domain containing 1; TRAIL, TNF-related apoptosis-inducing ligand; PKM2, pyruvate kinase isozyme M2.

**Table 2:** Possible therapeutic targets for IIMs.

Targets	Interventions	Death pathway	Year	References
**Aβ/macroautophagy**	Resistance exercise	Apoptosis, autophagy	2017, 2019	(42, 70)
**FAP**	Exercise and AMPK	Apoptosis	2020	(45)
**Calpain**	Calpeptin	Apoptosis	2010, 2011	(31, 46)
**Cathepsin B**	CA-074Me	Apoptosis	2013, 2015	(47, 48)
**mTOR**	Rapamycin	Autophagy	2017	(13)
**Heat Shock Factor-1**	Arimoclomol	Autophagy	2016	(69)
**AMPK**	IVIG	Autophagy	2018	(74)
**NLRP3**	Glyburide	Pyroptosis	2021	(102)
**P2X7 receptor**	Brilliant blue G	Pyroptosis	2021	(102)
**PKM2**	Shikonin	Pyroptosis	2021	(103)

Aβ, β-amyloid; FAP, fibro-adipogenic progenitor; AMPK, AMP-activated protein kinase; mTOR, mammalian target of rapamycin; IVIG, intravenous immunoglobulin; P2X7, adenosine triphosphate gated cationic channel; PKM2, pyruvate kinase isozyme M2.

## Author Contributions

JS collected materials and wrote the paper. QW, MT, SZ, and CW provided the idea and reviewed the manuscript. DX, JZ, XT, ML, and XZ helped with the final revision of the paper. All authors contributed to the article and approved the submitted version.

## Funding

This work was supported by the Beijing Municipal Science and Technology Commission (Z201100005520025), CAMS Innovation Fund for Medical Sciences (CIFMS) (2019-I2M-2-008), and the National Natural Science Foundation of China (81471615, 81601430).

## Conflict of Interest

The authors declare that the research was conducted in the absence of any commercial or financial relationships that could be construed as a potential conflict of interest.

## Publisher’s Note

All claims expressed in this article are solely those of the authors and do not necessarily represent those of their affiliated organizations, or those of the publisher, the editors and the reviewers. Any product that may be evaluated in this article, or claim that may be made by its manufacturer, is not guaranteed or endorsed by the publisher.
